# Concurrent training associated with moderate walnut consumption improved isokinetic strength, subjective sleep quality, cognitive performance and postural balance in elderly active men: a randomized controlled trial

**DOI:** 10.1007/s40520-023-02646-x

**Published:** 2024-02-29

**Authors:** Anis Kamoun, Abdelmonem Yahia, Mohamed Amine Farjallah, Rami Maaloul, Houssem Marzougui, Mohamed Bouaziz, Nizar Souissi, Mohamed Habib Elleuch, Omar Hammouda

**Affiliations:** 1 High Institute of Sport and Physical Education, University of Sfax, Sfax, Tunisia; 2 Research Laboratory of Evaluation and Management of Musculoskeletal System Pathologies, University of Sfax, LR20ES09 Sfax, Tunisia; 3 Research Laboratory, Molecular Bases of Human Pathology, LR19ES13, Faculty of Medicine, University of Sfax, Sfax, Tunisia; 4 Laboratoire d’Electrochimie Et Environnement, ENIS, Université de Sfax, LR14ES08, Sfax, Tunisia; 5 Research Unit: Physical Activity, Sport, and Health, UR18JS01, National Observatory of Sport, Tunis, Tunisia; 6 Interdisciplinary Laboratory in Neurosciences, Physiology and Psychology: Physical Activity, Health and Learning (LINP2), UFR STAPS, UPL, 200 Avenue de la République, 92000 Nanterre, France; 7 Université Sorbonne Paris Nord, Hypoxie et Poumon, H&P, INSERM, UMR 1272, Bobigny, F-93000 France; 8 Département STAPS, Université Sorbonne Paris Nord, Bobigny, France

**Keywords:** Aging, Walnut enriched diet, Combined training, Isokenetic strength, Cognitive performance

## Abstract

**Aims:**

To investigate the effects of concurrent training (resistance and endurance) associated with moderate walnut consumption on isokinetic strength, subjective sleep quality, cognitive performance and postural balance in physically active elderly men.

**Methods:**

Twenty healthy elderly men were divided into two matched groups, in a randomized controlled experiment. They have participated in three training sessions per week: concurrent (strength and endurance) training + ad libitum diet with walnuts (15 g/day for 6 weeks, CTW: *n* = 10) and concurrent training + ad libitum diet (CT: *n* = 10). Isokinetic strength, Spiegel questionnaire, Montreal cognitive assessment and postural balance parameters were assessed 48 h pre- and post-intervention.

**Results:**

Absolute peak torque of knee extensors and knee flexors significantly increased compared to pre-training in CTW (15.2% ± 6.7; 13.2% ± 2.3, *p* < 0.05, respectively) and CT (10.6% ± 6.8; 7.4% ± 2.9, *p* < 0.05, respectively). Subjective sleep quality increased compared to pre-training for CTW and CT (24% ± 14.4; 10.5% ± 9.4, *p* < 0.05, respectively) with a significantly greater increase in CTW (*p* < 0.05). Cognitive performance measured by Montreal cognitive assessment (MoCA) increased only in CTW compared to baseline (7.7% ± 2.5, *p* < 0.05). Postural balance parameters with dual task decreased only in CTW compared to baseline.

**Conclusions:**

The present study clearly revealed that concurrent training alone or associated with daily walnut (15 g) consumption for 6 weeks significantly increased knee isokinetic strength, support leg standing parameters and sleep quality. Meanwhile, cognitive performance evaluated by MoCA test and postural balance with dual task were improved for CTW group only.

## Introduction

Aging is a delicate period characterized by a deterioration of physical and mental capacities in humans [1, 2]. The ability of aging men to perform daily life activities is strongly related to the maintenance of the main elements of health-related physical fitness (i.e., strength production, muscle mass and balance control) [3, 4]. Aging is also characterized by a decline in visual, somato-sensory [5], vestibular [6] and neurobiological systems [7]. Cognitive impairment, sensory input deterioration and muscle weakness are common factors that seem to be primarily responsible for age-related gait disorders [8]. These complications cause postural instability and possible balance problems [9]. Interestingly, gait instability and falling risk increase upon shared attention or dual tasks (e.g., walking while talking) [10]. The daily multi-task situations are prone to dramatically impair autonomy and increase functional capacity dependency in the elderly [11].

Concurrent training (CT) involving strength and endurance exercises performed during the same session has been suggested to be a more effective strategy than performing only endurance or strength training only [12]. This training type has been proved to impact multiple components of fitness and health [4, 12]. Observational data support this assertion as older adults who meet both endurance and muscle-strengthening activity guidelines show a significantly better muscular and functional fitness performance [13].

Nutritional strategies can amplify the beneficial effect of CT in the elderly. It has been demonstrated that walnut dietary intervention improved lipid profile, steroid hormones and systematic inflammation in aged men performing concurrent training [14]. Otherwise, dietary intervention studies without physical training have shown preventive and therapeutic effects of walnut on age-related motor and cognitive deficits [15–17]. Likewise, it has been clearly demonstrated that long-term walnut supplementation in the diet significantly improves memory, learning skills, motor coordination, in mice model [16, 18, 19]. Indeed, walnut contains significant amounts of polyunsaturated fatty acids (PUFA) especially linoleic acid (LA) (18:2n-6) and alpha-linolenic acid (ALA) (18:3n-3) [18, 19]. Previous studies have shown that these essential fatty acids regulate many cerebral cellular processes, including learning and memory in the brain [18–20]. This nutriment is also one of the main food sources of phytomelatonin, hormone recognized as a sleep regulator with an average content of 350 ng/100 g [21].

Therefore, the purpose of this study was to examine whether walnut consumption amplifies or not the beneficial effect of CT on strength parameters, sleep quality, cognitive performances and postural balance in physically active elderly men.

## Methods

### Participants

Forty-six physically active elderly men (≥ 65 years) volunteered to participate in this study. Smoking, alcohol consumption and history of systematic physical training during 2 months before the study were exclusion criteria. No participant was taking drugs that influence the sleep/wake cycle. After receiving a thorough description of the protocol, its benefits and risks, each participant provided a written informed consent and underwent a clinical examination which included a full medical check-up, anthropometric measurements, resting electrocardiogram (ECG) and a 6-min walk test (6MWT). Twenty-eight participants meeting the participation criteria were divided into two groups in a randomized controlled trial: CT + ad libitum diet with walnut (CTW, *n* = 15); CT + ad libitum diet (CT, *n* = 13). Eight participants were withdrawn from data analysis due to protocol violations: acute infection with the use of antibiotics > 6 days (*n* = 4), personal reasons (*n* = 4) (i.e., obliged to leave the city due to family commitments). Ultimately, 20 men completed pre- and post-intervention (CTW, *n* = 10; CT, *n* = 10). The flowchart of participants was previously presented [14]. The baseline anthropometric measurements and 6 MWT of the participants are shown in Table 1.

**Table 1:** Baseline characteristics of the study participants

Variables	CT (M ± SD)	CTW (M ± SD)	*P*
Age (y)	66.9 ± 2.13	66.5 ± 2.68	0.71
Height (m)	1.71 ± 0.07	1.73 ± 0.06	0.62
Body mass (kg)	74.72 ± 7.95	73.09 ± 8.18	0.96
BMI (kg/m^2^)	25.51 ± 2.48	24.5 ± 2.45	0.72
6MWT (m)	216 ± 15.00	213 ± 19.78	0.82
% of total fat mass	22.2 ± 2.40	23.1 ± 2.10	0.82
% of lean mass	38.54 ± 1.62	37.94 ± 1.75	0.84

*CT* training group, *CTW* training + walnut, *M ± SD* mean ± standard deviation, *BMI* body mass index, *6MWT* 6-min walk test, *p* > 0.05 = no significant difference from CT

### Experimental design

**Fig. 1:**
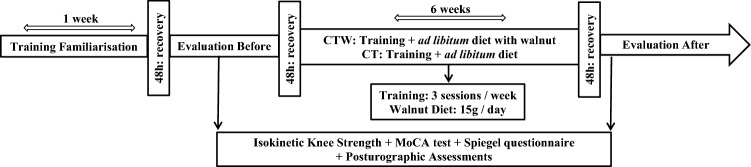
Study design

The experimental protocol consisted on two test sessions, 48 h, pre- and post-concurrent strength and endurance training (three sessions/week) during 6 weeks associated with a walnut supplementation for CTW (15g/day) only (Fig. 1). The concurrent program was previously described [14]. Body mass, % total fat and % lean body mass were measured on a professional impedance scale from Tanita (BC 418MY). According to the World Health Organization, body mass index (BMI) is calculated as the ratio of the weight measured in kilograms (Kg) by the square of the size measured in meters and is expressed in (Kg/m^2^).

Every participant underwent the following tests: isokinetic knee strength [22], maximal strength assessment [23], Spiegel sleep questionnaire [24], Montreal cognitive assessment (MoCA) [24] and balance tests containing several tasks [26]. One week prior to and during the experimental period, participants were instructed to refrain from consuming any type of walnut, except for the walnut provided, as well as to refrain from the use of aspirin and other over-the-counter pain relievers. The dietary intervention was previously described [14].

To evaluate the short-term (6 weeks) effects of CT and CTW groups, assessments were made prior to the initiation of daily walnut supplementation and at the end of the 6-week training period. Before starting the protocol, participants were familiarized with training exercises during 1 week (Fig. 1). They were requested to avoid physical activity for 48 h preceding each test.

### Isokinetic knee strength

The isokinetic knee flexor and extensor peak torque during concentric contraction  was measured at different angular velocities (120°.s^−1^ and 240°.s^−1 ^) using a Cybex Norm II Isokinetic Cybex® Norm II Medimex. Every participant was seated with hips and thighs firmly strapped to the seat of the dynamometer, with the hip angle at 85°. After that, a warm-up of 15 submaximal repetitions at 120°.s^−1^ was performed. Then, maximal sets of five repetitions at 120°.s^−1^ with a five min interval between the sets was taken. The contraction with the highest torque value was used in the data analyses. The test–retest reliability coefficients (ICC) were over 0.95 for both velocities. The total work of knee strength was measured with 20 repetitions at 240°.s^−1^ [22].

### Submaximal strength test

Maximal strength (1-RM) was estimated using the 10-RM approach [23]. Participants first performed a light warm-up in leg press, leg extension, leg curl, seated row and bench press machines. Load was progressively increased until participants could perform 10 or fewer repetitions [23]. The predicted 1-RM strength values were initially used to set the exercise load for the resistance training protocols.

### Spiegel sleep questionnaire

Subjective sleep quality was evaluated by the Spiegel sleep questionnaire [24]. This test includes six questions to judge the quality of sleep. It calculates a score that ranges from 0 to 30. The higher the score, the better the sleep quality [24].

### Montreal cognitive assessment (MoCA)

Montreal cognitive assessment (MoCA) is a rapid screening instrument for evaluation of cognitive performance [25]. It assesses different cognitive domains: attention, concentration, executive functions, memory, language, visuo-constructional skills, conceptual thinking, calculations, and orientation. The time to administer the MoCA is approximately 10 min. The total possible score is 30 points; a score of 26 or above is considered normal [25].

### Posturographic assessments

Postural balance was assessed using a stabilometric platform (SATEL®, Blagnac, France, 40 Hz frequency) in the laboratory of physical medicine. Postural orientation was assessed by measuring the displacement of the oscillation of the foot pressure center (center of pressure (CoP) CoP_x_: stability in the medio-lateral axis; CoP_y_: stability in the anterior–posterior axis).

Each participant underwent a static bipedal balance with a dual task and a support leg standing balance in two conditions “eyes open (EO)” and “eyes closed (EC)”. For each balance test, the average of three acquisitions was calculated **(**Fig. 1**)** [26]. Posturographic analysis was performed using a flat, rigid plate resting on three force sensors. Each participant was instructed to stand as still as possible on the platform and look horizontally (there was no particular visual target). The recording was started once the participant was ready. The measurement period lasted 51.2 s. A cognitive task was added for bipedal static evaluation in EO and EC conditions. This task consists of a count starting from 0 with an increment of 7 during measurement period.

### Concurrent training program

The strength exercises were performed first and were immediately followed by the endurance training [27]. CTW and CT groups performed the same exercise intensity and volume per session [27]. The strength training program included six exercises (leg press, leg extension, leg curl, seated row, bench press, and abdominal exercises). During the first 2 weeks, participants performed three sets of 12–10 RM, progressing to 10–8 RM (weeks 3–4) to finish with three sets of 8–6 RM (weeks 5–6). During each set, the workload was adjusted when repetitions performed were either under or above the repetitions established [28]. Endurance training session lasted 30 min with intensity individually monitored and maintained between 65% and 75% of the theoretical maximum heart rate [27].

### Dietary intervention

Walnut were purchased (Juglans regia) and distributed to participants. Participants of the CTW group were asked to consume 15 g of walnut at 10:00 am daily additionally to their habitual diets [29]. Moreover, participants were reminded by phone to consume walnut every day. No other specific dietary advice was provided and there were no restrictions on fat or calorie intake. For the CT group, participants were asked to maintain their usual dietary habits during the period of intervention. Before starting the protocol, the fatty acid compositions of the walnut samples were determined in the Laboratory of Electrochemistry and Environment, Sfax, Tunisia. Data are presented in Table 2.

**Table 2:** Lipid composition of walnut consumption

Nutrients 100 g	
**Lipids (g)**	**65.44**
**Polyunsaturated fatty acids (g) **	
*Linoleic acid (18:2n_6) (g)*	*39.62*
*α-Linolenic acid (18:3n_3) (g)*	*8.8*
**Monounsaturated fatty acid**	
*Oleic acid (18:1n_9) (g)*	11.1
**Saturated fatty acid**	
*Palmitic acid (g)*	4.15

### Statistical analyses

The statistical analyses were performed using the Statistica 10 software (StatSoft, Maisons-Alfort, France). Data were presented in the text, tables and figures as Mean ± Standard Deviation (M ± SD). Normality of the distribution was checked and confirmed using the Shapiro–Wilk test. The protocol-related effects were assessed using a two-way mixed Analysis of Variance (ANOVA) [(Group (CT, CTW) × Training (Pre, Post)] with repeated measures for training effect only. When ANOVA showed a significant effect, a Tukey *post-hoc* test was applied. Effect sizes were calculated as partial eta-squared ${\upeta }_{p}^{2}$ to assess the practical significance of findings of the present study. Statistical significance was set at *p* < 0.05.

## Results

### Isokinetic knee strength parameters and maximal strength assessment

The statistical analysis showed a significant effect of training for peak torque of knee extensor and knee flexor (F_(1,18)_ = 37.2, *p* < 0.001, ${{\varvec{\upeta}}}_{{\varvec{p}}}^{2}$ = 0.6; F_(1,18)_ = 41, *p* < 0.001, ${{\varvec{\upeta}}}_{{\varvec{p}}}^{2}$ = 0.7, respectively) (Table 3). The post hoc test showed that peak torque of knee extensor and flexor increased in the post-intervention for both CTW and CT groups (*p* < 0.05).

**Table 3:** Effect of concurrent training associated with moderate walnut consumption on strength parameters

Variables	Groups	Time		Anova		
		Pre (M ± SD)	Post (M ± SD)	Training Effect F_(1,18)_, *p*, ηp^2^	Group Effect F_(1,18)_, p, ηp^2^	Interaction F_(1,18)_, *p*, ηp^2^
Peak Torque flexors (N.m)	CT CTW	57.1 ± 7.85 49.6 ± 10.82	61.1 ± 7.25^*^ 55.6 ± 9.75^*^	41.2, <0 .001, .7	NS	NS
Peak Torque extentors (N.m)	CT CTW	89.8 ± 15 89.1 ± 19.78	99 ± 15.26^*^ 101 ± 17.99^*^	37.9, <0 .001, .6	NS	NS
Work flexors (J)	CT CTW	35.4 ± 5.48 33.5 ± 8.95	41.5 ± 6.9^*^ 41 ± 9.6^*^	37, < 0.001, .6	NS	NS
Work extensors (J)	CT CTW	63.4 ± 14.21 61.9 ± 16.13	74.5 ± 12.51^*^ 74.7 ± 14.46^*^	53, < 0.001, .7	NS	NS
1-RM Leg extension (kg)	CT CTW	37.9 ± 3.48 34.4 ± 6.98	55.4 ± 9.69^*^ 49.3 ± 10.55^*^	100, < 0.01, 0.85	NS	NS
1-RM Leg curl (kg)	CT CTW	20.8 ± 1.81 23.3 ± 4.11	31.3 ± 4.42^*^ 30.1 ± 6.05^*^	99.7, < 0.01, 0.84	NS	4.5, < 0.05, 0.2
1-RM Bench press (kg)	CT CTW	42.2 ± 6.76 42.2 ± 13.48	58.7 ± 8.54^*^ 54.1 ± 13.84^*^	227, < 0.01, 0.92	NS	5.9, < 0.05, 0.2

*CT* training group, *CTW* training + walnut, *Pre* Before intervention, *Post* 48 h after intervention, *M ± SD* mean ± standard deviation, *significant difference from pre-intervention (*p* < 0.05)

Concerning total work of knee strength, statistical analyses showed a significant effect of training for knee extensor and knee flexor (F_(1,18)_ = 53, *p* < 0.001, ${{\varvec{\upeta}}}_{{\varvec{p}}}^{2}$ = 0.7; F_(1,18)_ = 37, *p* < 0.01, ${{\varvec{\upeta}}}_{{\varvec{p}}}^{2}$ = 0.6, respectively) (Table 3). The post hoc test showed that total work of knee extensor and flexor increased inthe post-intervention protocol for both groups (*p* < 0.05).

Concerning maximal strength assessment, the two-way ANOVA showed a significant effect of training for leg extension (F_(1,18)_ = 100, *p* < 0.01, ${{\varvec{\upeta}}}_{{\varvec{p}}}^{2}$ = 0.85) ${{\varvec{\upeta}}}_{{\varvec{p}}}^{2}$ (Table 3). Leg curl and bench press exercises analyses showed a significant effect of training (F_(1,18)_ = 99, *p* < 0.001, ${{\varvec{\upeta}}}_{{\varvec{p}}}^{2}$ = 0.8; F_(1, 18)_ = 227, *p* < 0.01, ${{\varvec{\upeta}}}_{{\varvec{p}}}^{2}$ = 0.9, respectively) with significant interaction (Training × Group) (F_(1,18)_ = 4.5, *p* < 0. 05, ${{\varvec{\upeta}}}_{{\varvec{p}}}^{2}$ = 0.2; F_(1,18)_ = 5.9, *p* < 0.01, ${{\varvec{\upeta}}}_{{\varvec{p}}}^{2}$ = 0.2, respectively (Table 3).

The post hoc test showed that muscle strength increased in the post-intervention with regard to the lower limb movements (knee extension and knee curl) as well as some upper limb movements (Bench press) for both CTW and CT groups (*p* < 0.01).

### Spiegel questionnaire and MoCA test

**Fig. 2:**
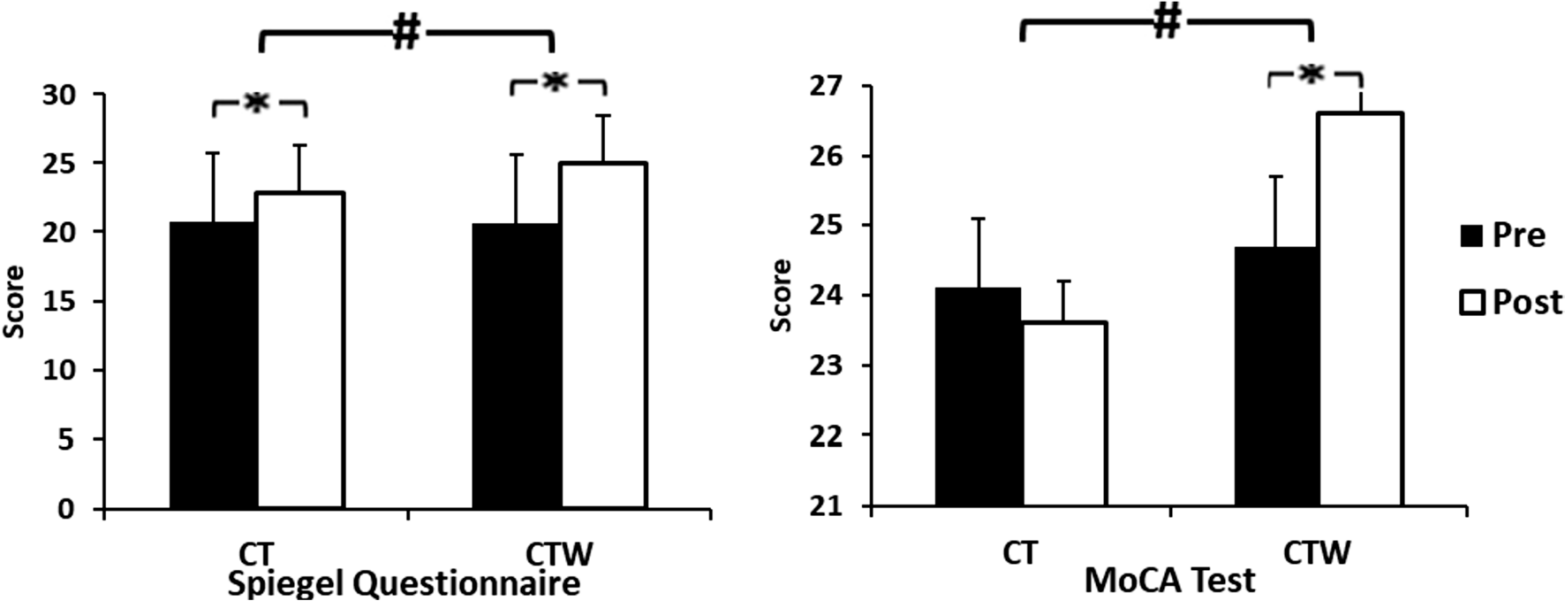
Effect of concurrent training associated with moderate walnut consumption on Spigel questionnaire and Montreal cognitive assessment (MoCA). *CT* training group + control diet, *CTW* training + walnut diet, *Pre* before intervention, *Post* 48 h after intervention, *M ± SD* mean ± standard deviation, *significant difference with pre-intervention (*p* < .05), #significant difference with CT (*p* < .05)

A significant effect of Training was observed for Spiegel questionnaire (F_(1,18)_ = 56, *p* < 0.001, ${\upeta }_{p}^{2}$= 0.7) with significant (Training × Group) interaction (F_(1, 18)_ = 6.7, *p* = 0.03, ${\upeta }_{p}^{2}$= 0.3) (Fig. 2). Spigel questionnaire increased post-intervention for both groups (*p* < 0.05). Moreover, it was higher for CTW group as compared to CT group.

Concerning MoCA test, there was a significant effect of Training for (F_(1,18)_ = 18, *p* < 0.05, ${{\varvec{\upeta}}}_{{\varvec{p}}}^{2}$ = 0.5) and Group (F_(1,18)_ = 14, *p* = 0.01, ${{\varvec{\upeta}}}_{{\varvec{p}}}^{2}$ = 0.4) with significant (Training × Group) interaction (F _(1,18)_ = 55, *p* < 0.001, ${{\varvec{\upeta}}}_{{\varvec{p}}}^{2}$ = 0.5) (Fig. 2). The *post-hoc* test showed that the MoCA test increase was greater in CTW comparatively with CT at post-intervention (*p* < 0.05).

### Posturographic parameters

#### Bipedal with dual-task parameters

Statistical analysis showed a significant effect of training (F_(1,18)_ = 27.2, *p* < 0.01, ${{\varvec{\upeta}}}_{{\varvec{p}}}^{2}$ = 0.6) with a significant (Training × Group) interaction (F_(1,18)_ = 9.4, *p* < 0.01, ${{\varvec{\upeta}}}_{{\varvec{p}}}^{2}$ = 0.3) for CoP_x_ in EO condition (Table 4). For CoP_y_, a significant effect of Training (F_(1,18)_ = 19.7, *p* < 0.01, ${{\varvec{\upeta}}}_{{\varvec{p}}}^{2}$ = 0.5) with a significant interaction (Training × Group) (F_(1,18)_ = 5.4, *p* < 0.01, ${{\varvec{\upeta}}}_{{\varvec{p}}}^{2}$ = 0.2) were found in EO condition (Table 3). The *post-hoc* test showed that CoP_x_ and CoP_y_ decreased in post-intervention protocol only for CTW (*p* < 0.05).

**Table 4:** Effect of concurrent training associated with moderate walnut consumption on bipedal with dual-task parameters

Variables	Groups	Times		Anova		
		Pre (M ± SD)	Post (M ± SD)	Training Effect F_(1,18)_, *p*, ηp^2^	Group Effect F_(1,18)_, *p*, ηp^2^	Interaction F_(1,18)_, *p*, ηp^2^
Cop_x_ BI EO (mm)	CT CTW	300.1 ± 110. 340.3 ± 147.8	276.6 ± 96.01 249.8 ± 103.3^*^	27.2, < 0.01, .6	NS	9.4, < 0.01, .34
Cop_y_ BI EO (mm)	CT CTW	419.6 ± 118.8 467.4 ± 125.1	419.1 ± 95.4 398.6 ± 101.0^*^	19.7, <0 .01, .5	NS	5.4, < 0.05, .24
Cop_x_ BI EC (mm)	CT CTW	304.5 ± 124.3 401 ± 190.63	291.0 ± 116.1 307.6 ± 127.2^*^	8.3, < 0.01, .3	NS	4.6, < 0.05, .2
Cop_y_ BI EC (mm)	CT CTW	524.9 ± 195.6 577.5 ± 162.4	501.2 ± 157.2 479.3 ± 124.3^*^	11.4 < 0.01, .4	NS	NS

*CT* training group, *CTW* training + walnut, *Pre* before intervention, *Post* 48 h after intervention, *M ± SD* mean ± standard deviation, *cop*_*x*_ center of pressure medio-lateral length, *cop*_*y*_ center of pressure antero-posterior length, *BI* bipedal, *EO* eyes open, *EC* eyes closed, *significant difference with pre-intervention (*p* < 0.05)

Concerning EC condition, analysis showed a significant effect of Training (F_(1,18)_ = 8.3, *p* < 0.01, ${{\varvec{\upeta}}}_{{\varvec{p}}}^{2}$ = 0.3) and a significant interaction (Training × Group) (F_(1,18)_ = 4.7, *p* < 0.05, ${{\varvec{\upeta}}}_{{\varvec{p}}}^{2}$ = 0.2) for CoP_x_ (Table 4). The two-way ANOVA showed a significant effect of Training (F_(1,18)_ = 47, *p* < 0.001, ${{\varvec{\upeta}}}_{{\varvec{p}}}^{2}$ = 0.6), a significant effect of group (F_(1,18)_ = 11.4, *p* < 0.01, ${{\varvec{\upeta}}}_{{\varvec{p}}}^{2}$ = 0.4) and a significant (Training × Group) interaction (F_(1,18)_ = 47, *p* < 0.001, ${{\varvec{\upeta}}}_{{\varvec{p}}}^{2}$ = 0.6) for CoP_y_ (Table 3). The *post-hoc* test showed that CoP_x_ and CoP_y_ decreased in post-intervention protocol for CTW only (*p* < 0.05).

#### Support leg standing parameters

**Fig. 3:**
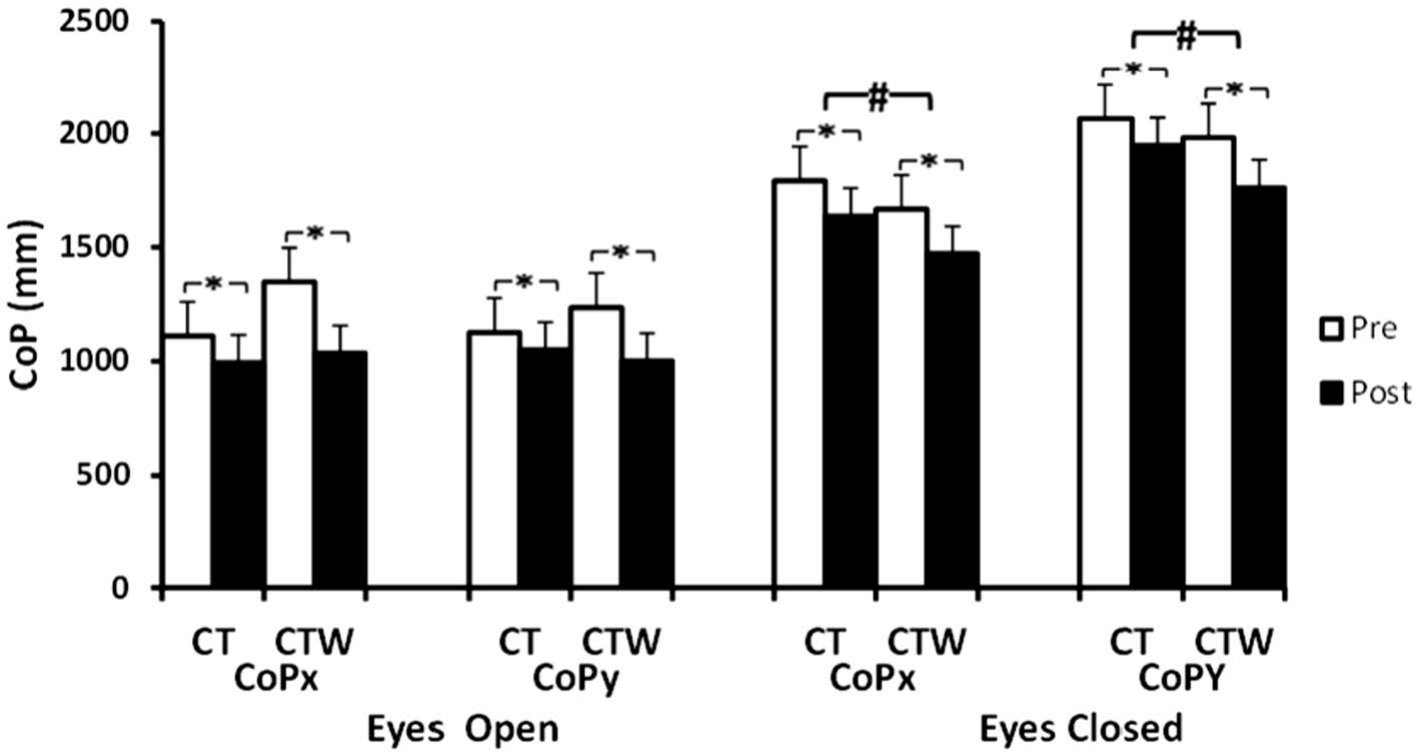
Effect of concurrent training associated with moderate walnut consumption on support leg balance parameters. *CT* training group + control diet, *CTW* training + walnut diet, *Pre* before intervention, *Post* 48 h after intervention, *M ± SD* mean ± standard deviation, *cop*_*x*_ center of pressure medio-lateral length, *cop*_*y*_ center of pressure antero-posterior length, *significant difference with pre-intervention (*p* < .05), ^**#**^significant difference with CT (*p* < .05)

Concerning the support leg standing, statistical analysis showed only a significant effect of Training, for CoP_x_ in EO (F_(1,18)_ = 17.5, *p* < 0.001, ${{\varvec{\upeta}}}_{{\varvec{p}}}^{2}$ = 0.5) and EC conditions (F_(1,18)_ = 24.8, *p* < 0.001, ${{\varvec{\upeta}}}_{{\varvec{p}}}^{2}$ = 0.5) (Fig. 3). Concerning CoP_y_, analysis showed a significant effect of Training (F_(1,18)_ = 16, *p* < 0.001, ${{\varvec{\upeta}}}_{{\varvec{p}}}^{2}$ = 0.4) with significant interaction (Training × Group) (F_(1,18)_ = 4.5, *p* < 0. 01, ${{\varvec{\upeta}}}_{{\varvec{p}}}^{2}$ = 0.2) in EO and significant effect of training (F_(1,18)_ = 40, *p* < 0.001, ${{\varvec{\upeta}}}_{{\varvec{p}}}^{2}$ = 0.5) and a significant interaction (Training × Group) (F_(1,18)_ = 5.7, *p* < 0.01, ${{\varvec{\upeta}}}_{{\varvec{p}}}^{2}$ = 0.2) in EC conditions (Fig. 3). The *post-hoc* test showed that CoP_x_ and CoP_y_ decreased post-intervention protocol for CT and CTW groups (*p* < 0.05) (Fig. 3).

## Discussion

The present findings showed that knee isokinetic parameters and muscle strength measured by 1 RM and isokinetic dynamometer were improved post-intervention compared to pre-intervention for both groups. In this sense, absolute peak torque of knee extensors and knee flexors was increased for CTW (15.2% ± 6.7; 13.2% ± 2.3, *p* < 0.05, respectively) and CT (10.6% ± 6.8; 7.4% ± 2.9, *p* < 0.05, respectively). Total work of the knee extensors and flexors was improved also for CTW and CT. The present findings showed also that the support leg standing parameters decreased for both groups in EO and EC conditions from baseline. These findings highlighted the beneficial effect of concurrent training on knee strength and support leg balance in aged men. Previous studies have reported that combined training improved isokinetic knee strength and support leg standing parameters in elderly [30–32]. This result could be due to the intrinsic factors associated to ageing, such as nervous and muscular system degenerative processes that lead to muscle weakness and gait instability [33]. In this sense, it has been demonstrated that a 6-week resistance exercise protocol improved strength, power and balance parameters in healthy older adults [31]. A similar result was reported in elderly women, showing that CT had larger increases in isokinetic strength than the aerobic training [24]. Furthermore, in the abovementioned study, the absolute peak torque increase in the CT group was greater comparatively with the aerobic training group, whereas the total work increased in both groups after training (*p* < 0.05) [24].

In congruence with the present study findings, it has been demonstrated that a concurrent aerobic and resistance training for 16 weeks improved one leg standing parameters and decreased the number of reported falls in older adults [30]. Another study reported that CT improved postural balance measures [31].

Otherwise, the present finding showed that Spiegel score (subjective evaluation of sleep quality) increased for CT and CTW by 24% ± 14.4 and 10.5% ± 9.4, respectively, with a greater increase of CTW. Studies investigating the effects of combined training associated with dietary supplements on sleep quality in aging remain scarce. However, it has been reported that regardless of the type of physical activity performed, regular mode rate exercise improves the sleep quality in the elderly [36–38]. Indeed, physical exercise alone possibly has a protective effect against the triggering of sleep disorders as part of non-pharmacological treatment [34]. Although combined training (strength/endurance) did not show superiority regarding sleep quality [35] compared to aerobic exercise alone [38], performing resistance exercise is important for the maintenance of functional capacity in elderly [38]. This combination is thus recommended for the elderly. Concerning the effect of walnut consumption alone, this nutriment is one of the main food sources of phytomelatonin, hormone recognized as a sleep regulator in humans, with an average content of 350 ng/100 g [21]. There is evidence that walnut consumption increased the serum melatonin levels in rats [21].

The present study highlighted that cognitive performance evaluated by the MoCA test improved by 7.7% ± 2.5 from baseline for CTW only. Likewise, bipedal postural balance with dual task was ameliorated in both conditions (i.e., EO, EC) only for CTW. This finding suggests a potential effect of walnut consumption on cognitive performance (memory) since bipedal balance was associated with cognitive task (a count starting from 0 with an increment of 7 during measurement period). Regarding the type of physical exercise, the effect of CT in this study confirmed a previous work that indicated no significant changes in cognitive performances after physical activities in aging men [39]. A possible explanation for the lack of cognitive benefit of the physical activity intervention is that the assigned level of physical activity may have been insufficient to produce changes in the cognitive measures despite its effect on physical function [40] and improvements in cognitive function in some shorter clinical trials [41]. In this sense, an increase in cognitive performances was recorded in older adults who exercised for a longer period (10 years**)** [24]. Concerning the isolated walnut effect on cognitive performance [42, 43], it has been demonstrated that walnut intake may be related to better overall cognition in aging, and could be an easily modifiable public health intervention [42, 43]. While most walnuts are high in monounsaturated fatty acids, they are unique because they are composed largely of PUFA, especially LA and ALA. Higher levels in PUFA have been shown to protect the brain from numerous insults associated with aging [15, 44] and may possibly allay age-related cognitive decline [14, 15, 44]. In fact, dietary supplementation with PUFA has been shown to slow age-related cognitive decline in humans. In a population-based prospective study in non-demented elderly subjects (65–84 years), it was found that high monounsaturated fatty acid and PUFA energy intakes were significantly associated with better cognitive performance on the Mini-Mental State Examination in 8.5-year follow-up assessment [45].

## Conclusion

The present study showed that concurrent training improved isokinetic strength and support leg standing parameters in trained aging men following or not a walnut enriched diet. We can conclude that concurrent training was proved to be a suitable nonpharmacological intervention which improved physical fitness and activities of daily living (i.e., reduced fall risks) in elderly. Moreover, subjective sleep quality was more improved in the CTW group than the CT one. Meanwhile, memory and postural balance with dual task were ameliorated only in the CTW group. This could be due to the beneficial effect of PUFA contained in walnut, since PUFA can stimulate molecular systems involved in neuronal functions and brain plasticity. Thus, short-term moderate walnut consumption could amplify the beneficial effect of concurrent training in the elderly.

## Limitation and perspective

The major limitation of the current study is a lack of dietary assessment for both groups, at baseline.

## Data Availability

The data that support the findings of this study are available from the corresponding author upon reasonable request.
